# Low-dose radiation from A-bombs elongated lifespan and reduced cancer mortality relative to un-irradiated individuals

**DOI:** 10.1186/s41021-018-0114-3

**Published:** 2018-12-19

**Authors:** Shizuyo Sutou

**Affiliations:** School of Pharmacy, Shujitsu University, 1-6-1 Nishigawara, Naka-Ku, Okayama-Shi, 703-8516 Japan

**Keywords:** A-bomb survivors, Lifespan, Life Span Study, Linear no-threshold, LNT, Longevity, Residual radiation, Threshold

## Abstract

The US National Academy of Sciences (NAS) presented the linear no-threshold hypothesis (LNT) in 1956, which indicates that the lowest doses of ionizing radiation are hazardous in proportion to the dose. This spurious hypothesis was not based on solid data. NAS put forward the BEIR VII report in 2006 as evidence supporting LNT. The study described in the report used data of the Life Span Study (LSS) of A-bomb survivors. Estimation of exposure doses was based on initial radiation (5%) and neglected residual radiation (10%), leading to underestimation of the doses. Residual radiation mainly consisted of fallout that poured down onto the ground along with black rain. The black-rain-affected areas were wide. Not only A-bomb survivors but also not-in-the-city control subjects (NIC) must have been exposed to residual radiation to a greater or lesser degree. Use of NIC as negative controls constitutes a major failure in analyses of LSS. Another failure of LSS is its neglect of radiation adaptive responses which include low-dose stimulation of DNA damage repair, removal of aberrant cells via stimulated apoptosis, and elimination of cancer cells via stimulated anticancer immunity. LSS never incorporates consideration of this possibility. When LSS data of longevity are examined, a clear J-shaped dose-response, a hallmark of radiation hormesis, is apparent. Both A-bomb survivors and NIC showed longer than average lifespans. Average solid cancer death ratios of both A-bomb survivors and NIC were lower than the average for Japanese people, which is consistent with the occurrence of radiation adaptive responses (the bases for radiation hormesis), essentially invalidating the LNT model. Nevertheless, LNT has served as the basis of radiation regulation policy. If it were not for LNT, tremendous human, social, and economic losses would not have occurred in the aftermath of the Fukushima Daiichi nuclear plant accident. For many reasons, LNT must be revised or abolished, with changes based not on policy but on science.

## Background

Japan is the only country that has sustained a nuclear attack. The weapons dropped in 1945 killed approximately 200,000 people instantaneously. People around the world have been taught for decades since that ionizing radiation is limitlessly hazardous, This supposition is based on a linear no-threshold model (LNT): even the lowest doses of ionizing radiation are hazardous in proportion to their doses, Therefore, it is quite natural that most people think that ionizing radiation from the A-bombs killed people, shortened lifespan, and increased cancer mortality. The Fukushima Daiichi nuclear power plant accident presented an opportunity to study the effects of ionizing radiation on health, after which the author published associated books [1, 2] and papers [3, 4]. Through their composition, it became increasingly clear that LNT has a seriously flawed history [5]. The energy of A-bombs comprised 35% thermal radiation (heat and light), 50% blast energy (pressure shock waves), and 15% nuclear radiation [6]. In fact, instantaneous deaths were mostly ascribable to thermal and blast energy (85%), especially in the central area of the blast. People tend to forget that victims of heat and blast were affected in a moment or short period, whereas cancer induction has remained a menace even to the present day. For survivors of today, fear of A-bombs mostly overlaps with fear of cancer. It is less well known that ionizing radiation is not always hazardous. Low-dose radiation sometimes stimulates our defense mechanisms and beneficial (radiation hormesis) [7–10].

Taking these facts into consideration, the effects on lifespan and cancer incidence of A-bomb survivors were reexamined for the present analyses. Letting the data speak, one would hear that low-dose radiation from A-bombs has extended survivor lifespan and reduced cancer mortality on average for A-bomb survivors and not-in-the-city control subjects (NIC). The key to resolving the apparent discrepancy between the received notions and actual data is radiation hormesis and the radiation doses of a hormesis range to which a large fraction of A-bomb survivors and NIC were exposed. Of course, A-bomb survivors who received high doses exhibited shortened lifespan and increased cancer mortality, but they accounted for a minor fraction of all local residents. Therefore, results show that the “average lifespan” was longer and that “average cancer mortality” was reduced overall.

Radiation units such as rem, Sv, and Gy are used here as reference articles use, unless otherwise specified.

## Longer lifespan of some people who were heavily irradiated by ionizing radiation

Reportedly the unhappiest man in the world, Mr. Tsutomu Yamaguchi, was A-bombed at Hiroshima. Later he relocated to Nagasaki, where he survived the second A-bomb attack [11]. He survived the two A-bomb attacks; he might be the happiest man in a sense that more than 70 people were evacuated from Hiroshima to Nagasaki: all except him were killed. More surprising is that the two A-bombs did not shorten his life: he died of stomach cancer at 93.

The Nikkei Shimbun reported on April 5, 2018 that Chairman Sunao Tsuboi of the Japan Confederation of A-Bomb and H-Bomb Sufferers Organizations was selected as an honorary citizen of Hiroshima City. When he was 20, the A-bomb attacks occurred when he was 1.2 km from the epicenter. He is 93 in 2018. He talked to then US President Obama to encourage efforts to abolish nuclear weapons. The occasion on May 27, 2017 was the first visit ever to Hiroshima by a serving president.

When he was 8, Shigeaki Mori was blown into a riverbed from a bridge and injured 2.5 km from the epicenter. He became a historian and discovered that American victims of the A-bomb were present in Hiroshima. His finding was reflected in President Obama’s speech, “Why do we come to this place, to Hiroshima? We come to ponder a terrible force unleashed in the not so distant past. We come to mourn the dead, including over 100,000 Japanese men, women and children, thousands of Koreans and a dozen Americans held prisoner.” After the speech, a tearful Mori was embraced by Obama. Born in 1937, he has lived longer than the average for Japanese men.

Dr. Don Wiles, Emeritus Professor of Chemistry at Carlton University, Canada, once engaged in extraction of radium from uranium ore for 16 months from 1947. Before the use of cobalt, radium ($20,000/g) encapsulated in a glass tube was used to treat cancer by embedding it into the malignant tissues. The crystallization process used by Marie Curie 50 years before included procedures that were apparently very lax and coarse compared to the present standard: encapsulation was performed with bare hands. Workers ignored the rule to wear rubber gloves because they were slippery. Radiation badges even under the lead shield became black at the daily check. Because radium is similar to calcium in terms of its chemical characteristics, radium was apparently accumulated in Dr. Wiles’ bones. Born in 1925, he exhaled about 25 times the legal maximum of radon, a product of radium, at the age of 88. One might assume that he was seriously injured. He stated “About 65 years later, I am still healthy.” [12]. These are some examples of increased longevity despite radiation exposure. Are they exceptional?

## A-bomb survivors lifespans are unusually long

**Fig. 1:**
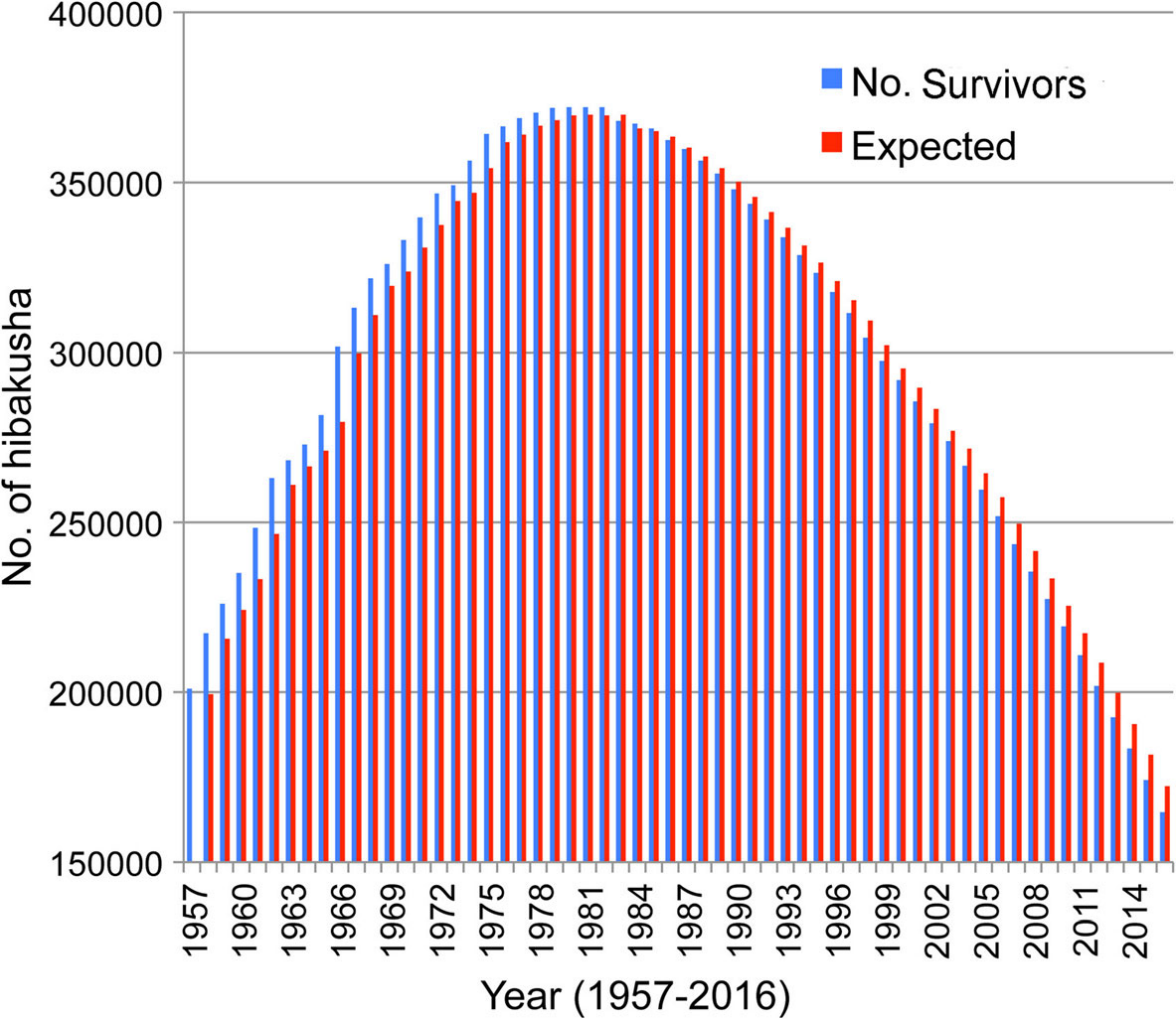
Changes of people who have an A-Bomb Survivor’s Certificates (Ministry of Health, Labour and Welfare [58] (blue). For example, a total of 183,519 certificate holders in 2014 comprised four classes: 1st class survivors, or direct victims (113,685); 2nd class survivors, or in-city victims who were within areas inside 2 km from the epicenter (42,529); 3rd class survivors, or rescue victims who engaged in rescue activities or physical treatments outside the 2 km areas and who were exposed to residual radiation (20,013); and 4th class survivors, or fetuses of people in one of the above three categories (7292). Their peak number was 372,264 in 1980. Expected numbers (red) were calculated as follows: holders in 1957 were 200,984; the death ratio of the Japanese in 1957 [59] was 0.008275 and 1663 (200,984 × 0.008275) were expected to die and 199,321 (200,984–1663) was the expected number in 1958 (the same hereinafter). Certificate holders are supported financially with six allowances and funeral fees. Some other benefits accrue: they can undergo free health examinations twice a year; and almost all sicknesses are treated at no charge. Patients with illness caused by a nuclear weapon were eligible to receive an allowance of 138,380 yen/m. The health control allowance is 34,030 yen/m. The funeral allowance is 206,000 yen. The total budget for fiscal year 2015 was 393,391,000,000 yen

Figure 1 presents changes of the number of certificate holders who have been covered by the Law Concerning Relief to Atomic Bomb Survivors. The holders are regarded as A-bomb survivors. Until 1982, holders were more than expected because additional people had been admitted as holders; the holders’ superiority in number does not necessarily mean that holders had a long lifespan. After 1982, the expected number became greater than the actual holders because few people were admitted as new and holders were getting steadily older year by year. The mortality ratio of the Japanese that was used to calculate the expected numbers was the average of infants, young people, adults, and elderly people, producing a result that is much less than that of the aged holders. Therefore, the holders’ exposure and experience do not necessarily mean that their lifespan is short.

The average lifespan of certificate holders was 80.13 for 2014. The ratio of men to women is not available. The lifespan of Japanese men was 80.21 for 2013 and that for women was 86.61; the average was 83.49. The life expectancy (for remaining life) at age 80 is 8.61 for men and 8.19 for women. Therefore, the lifespan of A-bomb survivors is expected to be over 88, far exceeding the average. This elongated average lifespan of holders might be ascribable to good medical services offered by the Japanese government. This might have contributed to some degree, but apparently some other important factor has an influence: low-dose radiation stimulates human biological defense mechanisms.

## A-bomb survivors lifespan was statistically shortened

Cologne and Preston investigated the longevity of 120,321 A-bomb survivors [13]. They concluded that “Median life expectancy decreased with increasing radiation dose at a rate of about 1·3 years per Gy, but declined more rapidly at high doses. Median loss of life among cohort members with estimated doses below 1 Gy was about 2 months, but among the small number of cohort members with estimated doses of 1 Gy or more it was 2·6 years. Median loss of life among all individuals with greater-than-zero dose estimates was about 4 months.” Almost all readers of the summary sentences above must believe that ionizing radiation from A-bombs was hazardous and that it shortened A-bomb survivors’ longevity to a greater or lesser degree. One must nevertheless be alert. The A-bomb survivors lifespan was not necessarily shortened, as described later. When a model cannot explain established facts, not the facts but the model must be wrong. What reasons are there in the discrepancy between actual life elongation and statistical shortening of lifespan? Apparently, three major factors engender wrong conclusions: 1) invalid LNT was promulgated – one never considers life elongation and cancer mortality reduction as effects of radiation; 2) a false assumption (zero exposure-zero risk) in NIC was used by neglecting residual radiation; and 3) radiation hormesis, the idea that low-dose radiation stimulates defense systems, was neglected. These three points are briefly examined before returning to discussion of Cologne and Preston’s data [13] later.

## LNT is not based on solid data

### Muller’s tenacity to maintain LNT

The origin of LNT dates back to 1927, when Muller found that X-rays induced sex-linked recessive lethality in *Drosophila melanogaster* [14]. This “data-poor/discussion-rich” paper was quite likely to have cleverly circumvented the normal peer review process [15]. Later, he presented related data. Apparent linearity at extremely high doses was extrapolated to lower doses without experimental data. He put forward the proportionality rule, an analog of LNT [16]. Then in 1939, World War II (WWII) broke out. The United States of America (USA) began production of the A-bomb under its Manhattan Project. Radiation effects on living organisms were investigated intensively. He learned of a threshold for positive excess risk in recessive lethality tests of *D. melanogaster* [17]. The US dropped A-bombs on Hiroshima and Nagasaki in 1945. Muller became a Nobel laureate in 1946 for his radiation research. Although he knew of thresholds to damage from radiation, he declared in his Nobel Prize lecture that there was “no escape from the conclusion that there is no threshold dose” [18].

### Oil industries felt uneasy about nuclear energy and took over the National Academy of Sciences

Standard Oil Co. Inc. was founded by John Rockefeller in 1870, who later established the Rockefeller Foundation (RF) in 1913. The oil industry might well have felt threatened by the discovery of atomic energy. The Republican Party had forged a close relationship with the oil industry, but the Democratic Party, led by F.D. Roosevelt (1933–1945) and H. Truman (1945–1953), governed the USA during and after WWII. When Republicans were reelected, Nelson Rockefeller was appointed as an important aide to President Eisenhower. Muller, in turn, had close ties to the RF. In 1954, the RF chose to finance a large project to evaluate ionizing radiation. RF asked the U.S. National Academy of Sciences (NAS) to organize the program, which was conducted under the auspices of NAS President Bronk of Rockefeller University, also an RF trustee. The Genetics Panel (GP) of the NAS Biological Effects of Atomic Radiation (BEAR) committee was established in 1954 and was chaired by Weaver, a mathematician and director of RF.

With no significant discussion, GP recommended LNT on June 12, 1956 [19]. The limit dose for nuclear workers of 500 mGy/y, which had been in place since 1934, was discarded. The next day, the front page of the New York Times, owned by an RF trustee, reported that radiation is dangerous. Other media followed suit. Soon, several leading biologists asked GP to provide documentation that supported LNT. GP refused to do so because they never possessed relevant data. This decision was cast, and reasonably so, as an ideologically motivated choice based on deliberate falsification and fabrication of research records [20]. Fossil fuel companies are opposed to nuclear energy even today.

### Expansion of LNT from insect sperm to the human body

Lewis (a 1995 Nobel laureate) argued in 1957 that radiation-induced leukemia conformed to the LNT hypothesis [21]. This was a new deployment of LNT from germ cells (heritable effects) to somatic cells (cancer induction). Several prominent researchers criticized the Lewis’ paper (Table 2 in ref. [22]). With no convincing data to support LNT reported for half a century, the Biological Effects of Ionizing Radiation committee of NAS published BEIR VII report in 2006 to support LNT [23]. This report includes several shortcomings, as discussed later. Moreover, LNT has been applied also to chemical carcinogens; the smallest amount of a carcinogen is hazardous without threshold for positive excess risk.

## Radiation doses are underestimated by neglecting residual radiation or black rain

### Residual radiation and the formation of black rain

The radiation doses for A-bomb survivors were estimated using radiation transport calculations based on radiation transport findings from tests conducted on the ground in the Nevada desert. The nuclear weapons dropped on Hiroshima and Nagasaki were detonated respectively at 600 m and 503 m heights. To obtain more accurate data, the ICHIBAN project was planned, for which a 510 m high tower was constructed in the Nevada desert [24]. A nuclear reactor or other radiation source was placed at the top of the tower and data were collected. The dosimetry of the ICHIBAN project was named tentative dose 1965 (T65D). Around the 1980s, results demonstrated that T65D did not correctly reflect A-bomb radiation intensity. Exposure doses were reexamined, after which the Dose System 1986 (DS86) was established. In the period around the 1990s, DS86 was revised again; Dose System 2002 (DS02) was established. DS02 was revised further as DS02R1, producing the current system used to estimate the exposure doses of A-bomb survivors [25]. Although dose systems have been revised several times, T65D is the basic one. Others are modified versions that do not deviate greatly from T65D. T65D was an outcome of a large-scale simulation model of A-bombs, but it included an important oversight, i.e., omission of residual radiation with a dose twice as large as the initial radiation on which the dose estimation was made.

**Fig. 2:**
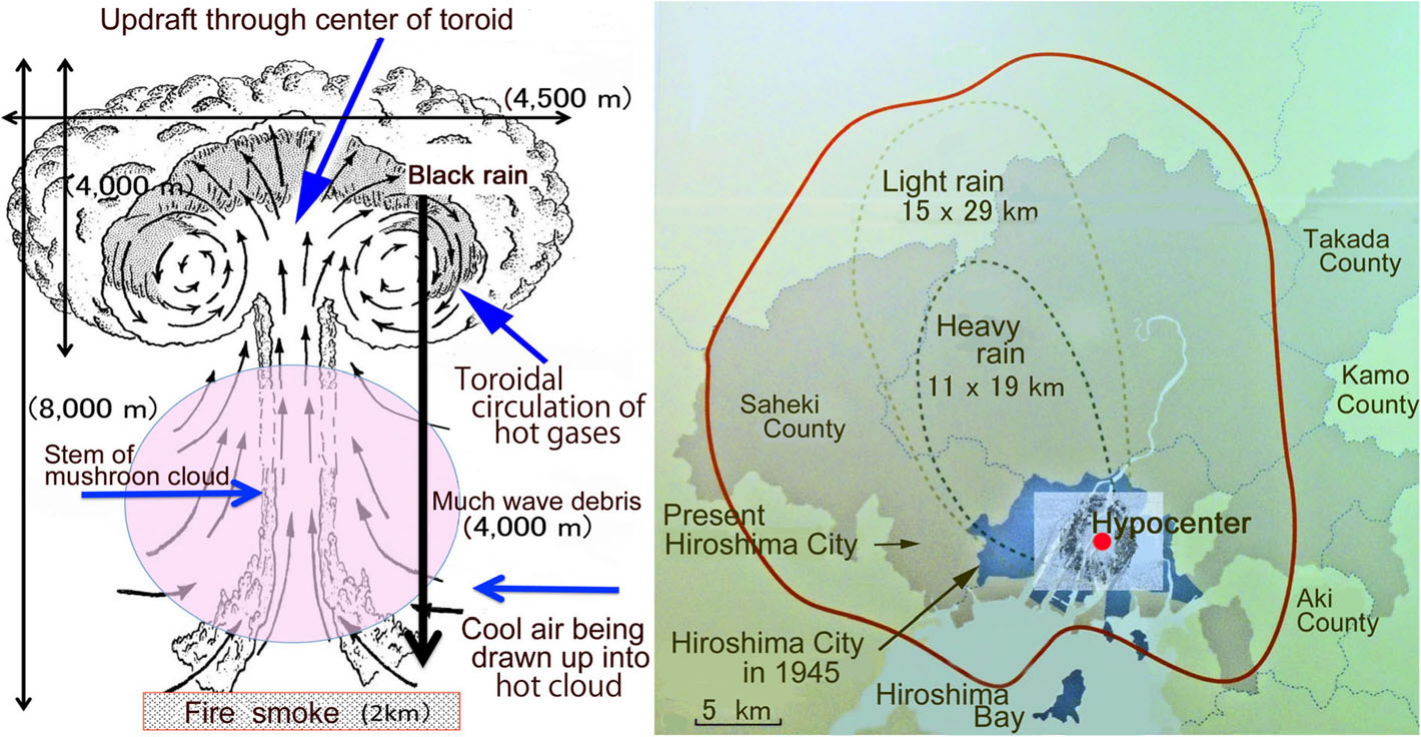
Formation of black rain from the mushroom cloud (left), and black rain areas (right) [27]. Left: The A-bombs used to attack Hiroshima (16 kt TNT equivalent) and Nagasaki (21 kt TNT equivalent) were detonated respectively at 600 m and 503 m heights. A 500-m diameter fireball is formed by the detonation of a 20 kt bomb. The fireball rose like a skyrocket. During expansion of the ball, vaporized matter was condensed to a doughnut-shaped cloud with violent internal circulatory motion. Following the rising fireball, dirt and debris were sucked up from the Earth’s surface. A Mach wave (the tip reaching 560 m 1.25 s after the blast) was reflected from the surface, whirling soil and debris up to form a Mach wave mass of 3800 t, producing black rain with raw materials together with the mushroom components. Trees, lumber, and other matter of 1.55 × 10^5^ t were incinerated, forming a smoky fire 2 km in diameter, above the ground. Two references [65, 66] were used to draw this figure. Right: The probable heavy rain area reported in 1953 is shown as a thick broken line. That of light rain is shown as a thin broken line. The black rain area according to analyses of the “A-bomb Survivors’ health awareness survey” in 2008 is shown as a solid red line (Hiroshima Peace Memorial Museum). A red circle off center denotes the epicenter. Black dots around the epicenter show locations of A-bomb survivors at the T65D survey [67]

The energy of a typical A-bomb comprises three components: 35% thermal radiation (heat and light), 50% blast energy (pressure shock wave), and 15% nuclear radiation [6]. Of that latter 15%, 5% is initial radiation (released within 30 s). The remaining 10% is residual radiation, which consists of major fallout and minor induced radioactivity. Induced radioactivity is produced by the action of neutrons in making non-radioactive substances into radioactive ones, but its lifespan is very short and is mostly negligible. A large fraction of the fallout, 40–70%, is believed to settle onto the ground within a day, but this depends strongly on weather and geographical features. When T65D was established, Black rain never fell in the Nevada desert. At Hiroshima and Nagasaki, thermal radiation incinerated or scalded plants, animals (including humans), houses, and various organic substances, producing heat, carbon dioxide, and vapor and consuming oxygen. Heat killed people. A lack of oxygen contributed deaths by suffocation. Victims were therefore affected in various ways by the A-bombs. From many waterways in Hiroshima and Nagasaki, large volumes of water were evaporated. The water itself was sucked up as if by a tornado. The vapor and water went up into the sky and cooled, thereafter forming raindrops containing soot and other debris. The resultant black rain started to pour down 20–30 min after the detonation. The rainfall lasted for a few hours (Fig. 2). The heavy black rain is well known to be highly radioactive. The possibility exists that the black rain included the most fallout, two-thirds of the nuclear radiation energy, i.e., twice as much radiation as the initial radiation used to estimate the radiation doses.

### Evidence that residual radiation fell to the ground with the black rain

**Fig. 3:**
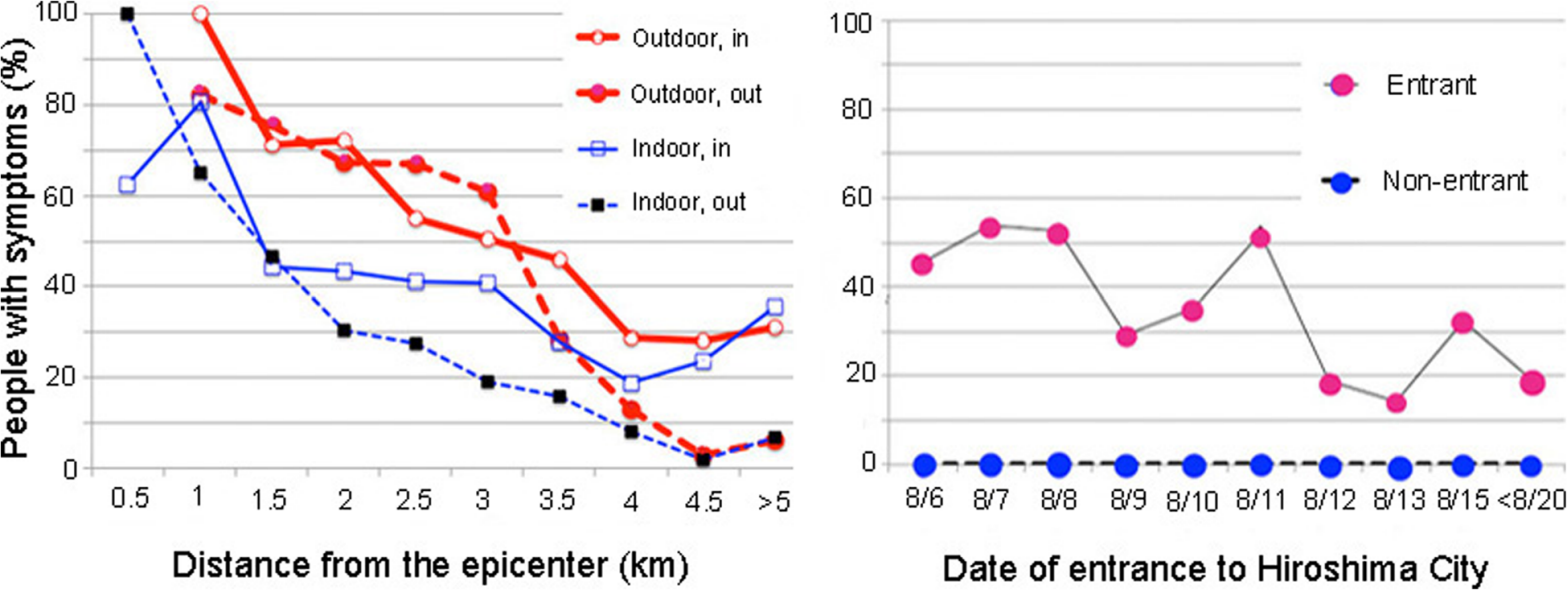
Proportion of A-bomb survivors with symptoms (left) and that of non-A-bomb survivors with symptoms (right) [27]: Left, open circles (Outdoor, in), outdoor A-bomb survivors who entered the central area; filled circles (Outdoor, out), outdoor survivors who did not enter the central area; open squares (indoor, in), indoor survivors who entered the central area; and filled square (indoor, out): indoor survivors who did not enter the central area. Right, red circles (entrant), non-A-bomb survivors who entered the central area and blue circle (non-entrant), non-A-bomb survivors who did not enter the central area

An old Japanese article written in 1957 by G. Obo [26] was later translated into English [27]. For the article, approximately 4000 people who lived in a 7 km radius from the epicenter were interviewed personally if they entered the central area 1 km radius from the epicenter and if they had radiation acute effects such as skin burn, external injury, fever, diarrhea, sore throat, skin bleeding, or loss of hair. Students of Hiroshima University took part in this study. Fundamentally important data are presented in Fig. 3.

The left panel of Fig. 3 shows 1) positive relations between people with symptoms and distance from the epicenter, 2) outdoor people as more severely affected than indoor people as a matter of course, 3) people in the areas ≥3 km from ground zero (beyond the reach of γ-rays and neutrons from initial radiation) were affected, implying that this area was contaminated severely by residual radiation most probably carried by black rain, and 4) indoor and outdoor people who were at a distance ≥4 km and who entered the central areas were affected almost equally independent of their distance from the epicenter, strongly suggesting effects of residual radiation. The right panel of Fig. 3 shows that a large fraction of non-A-bomb survivors entered the central area 2–3 weeks after detonation suffered from severe radiation sickness as if they were A-bomb survivors. This result indicates strongly that the area was heavily contaminated with residual radiation associated with black rain.

### Report that black rain is negligible is refutable

The effects of black rain were studied using mortality data from 1950 to 2005 and cancer incidence data from 1958 to 2005 in Hiroshima and Nagasaki. The authors conclude that deleterious health effects from black rain exposure were not detected [28]. However, there is apparently a methodical fault. The authors asked people, “Was the person caught in Fallout Rain?” (Yes or No). According to the response, they were then divided into Yes or No groups. This grouping is almost meaningless because the important matter is not Yes or No, but if they had entered black rain affected areas within 2–3 weeks after detonation when residual materials remained active (Fig. 3). When solid cancer deaths and solid cancer incidence are extracted from the literature [29], excess relative risks (ERR) were smaller in the Yes group (caught in the rain) than in the No group (not caught in the rain) (Table 1). The data are suggestive of hormesis: slight radiation exposure is cancer-inhibitory.

**Table 1:** Excess relative risks for exposure to black rain for solid cancer death and solid cancer incidence (solid cancer incidence for 1950–2005 and solid cancer death for 1958–2005 were not available)

Data	Fallout rain status	No. of cases	Excess relative risk (ERR)
1962–2005			
Solid cancer death	No	3573	0.00
	Yes	1483	− 0.04
Solid cancer incidence No	5653	0.00	
	Yes	2283	−0.06
1950–2005			
Solid cancer death	No	3970	0.00
	Yes	1633	−0.02
1958–2005			
Solid cancer incidence	No	5982	0.00
	Yes	2430	−0.03

The black rain affected areas were so wide that almost all A-bomb survivors and NIC must have been irradiated to a greater or lesser degree by residual radiation. The UNSCEAR 1958 report describes that almost all leukemia patients in zone C (1500–1999 m from ground zero) complained of severe radiation sickness in spite of an estimated dose of 50 rem (500 mSv in the International System of Units (SI)). Their doses must have been greater than 50 rem [30]. Exposure of around 2 Gy (close to 2 Sv in SI) is necessary to induce severe radiation sickness.

## BEIR VII report fails to support LNT

### BEIR VII report, the second problematic assertion by the National Academy of Sciences

**Fig. 4:**
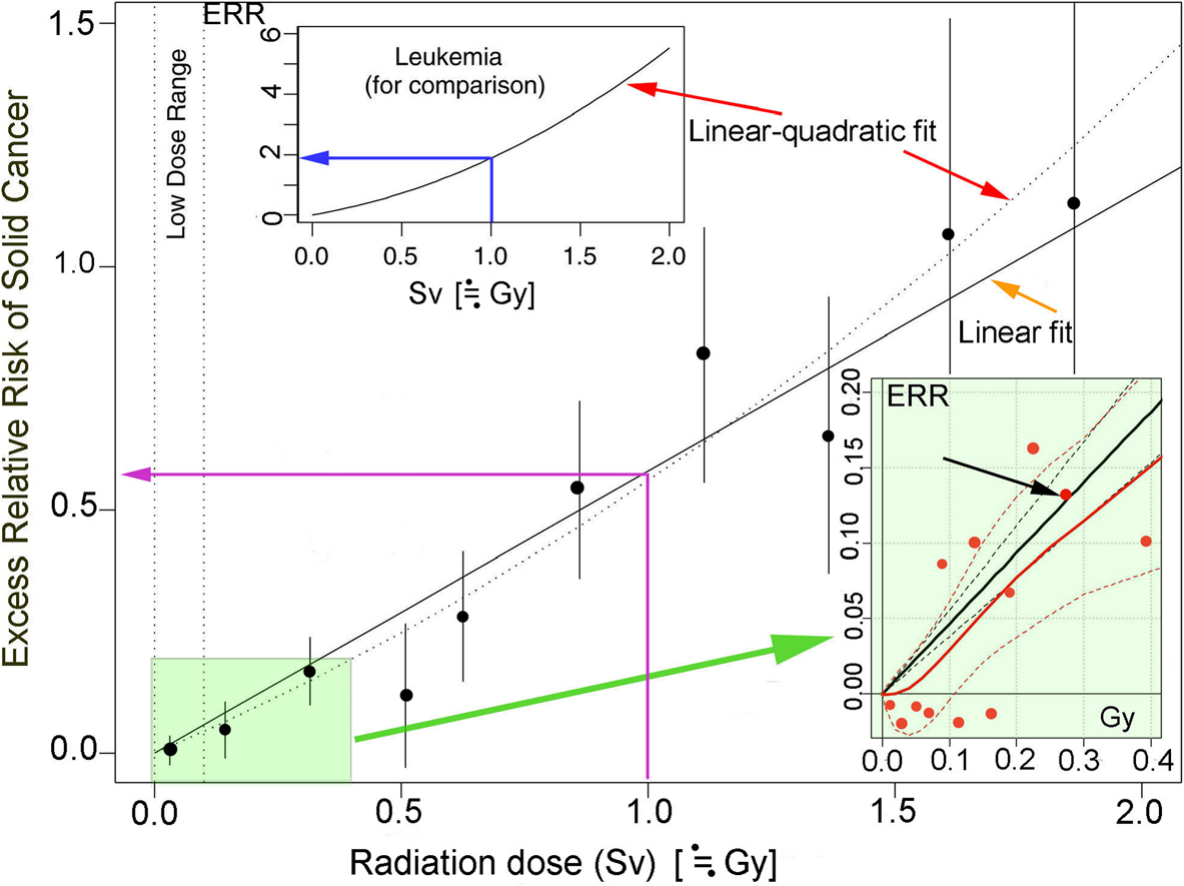
Excess relative risk (ERR) of solid cancers for Japanese A-bomb survivors [23]. The plotted vertical lines represent approximate 95% confidence intervals. The thin blue, purple, red, and orange arrows respectively indicate ERR/Sv for leukemia, ERR/Sv for solid cancer, linear quadratic fit, and linear fit. The lower left area in pale green is enlarged as the lower right insert, which shows the results of two statistical analyses [35]. The black straight and dotted lines respectively show the linear fit of LNT and 95% confidence intervals. The red continuous and dotted lines respectively show the Bayesian fit and 95% credible interval. The black arrow indicates only one dot inside the 95% confidence intervals. Less than 100 mSv constitutes the low-dose range

Originally, LNT was based on Muller’s experiments using repair-deficient *Drosophila* sperm [14]. He knew of the existence of thresholds for positive excess risk in *Drosophila* tests [17]. Indeed, later experiments by Japanese researchers indicate clearly that *Drosophila* irradiated with X-rays [31] or γ-rays [32] show not only thresholds but also hormesis. Hormesis has been observed in A-bomb survivors for solid cancers [29] and leukemia [33]. In spite of a large body of experimental data against LNT, NAS, the founder and advocator of LNT since 1956 [19], presented the BEIR VII report as basic LNT-supportive data (Fig. 4) [23]. The support, based on a Life Span Study (LSS) of A-bomb survivors, has been regarded as the gold standard to estimate radiation risk for human cancer. Nevertheless, this analysis presents serious flaws as explained below.

By the way, both Sv and Gy units are used according to original references in Fig. 4. Sv is a suitable unit for LNT and more generally acceptable Gy is used in this chapter.

### Leukemia, a better indicator of radiation stochastic effects than solid cancer

Leukemia, a cancer of the blood cells, is a better indicator of radiation than problematic solid cancers because it is sensitive to radiation. It appears around 2 years after exposure and reaches a peak 6–8 years later, whereas solid cancers start to appear around 10 years after exposure and last for decades. Figure 4 (upper left insert, blue arrow) shows that ERR/Gy for leukemia is approximately 2, whereas that for solid cancer is approximately 0.55 (lower left, purple arrow). Therefore, leukemia is sensitive to radiation and a better indicator than solid cancers. The dose-response of leukemia is not linear but is instead linear-quadratic (upper left insert). That of solid cancer also fits better to linear-quadratic (red arrow) than linearity (orange arrow), but no statistical significance was found between the two; BEIR VII asserts linearity. This forcible logic is difficult to accept. Moreover, when taking into consideration neglected residual radiation, effects of blast/thermal wave injury on the immune system, and hormesis, dose responses might be deviated far from linearity.

### Concealment of downturn

When radiation doses are much higher than 2 Gy, exposed people tend to die of adverse effects before reaching an age when cancer commonly occurs; ERR would show a downturn until finally reaching zero. The highest dose in Fig. 4 is 2 Gy, which conceals the downturn. Indeed, “The dose-response curve shows some downward bending in the high-dose range (2 + Gy in organ dose) for leukemia and even for all cancer except leukemia” [34]. When following the dots in Fig. 4 from low to high with downturn over 2 Gy in mind, one can easily imagine a sigmoid-like curve. This is shown by Bayesian analysis of LSS (Fig. 4, right below insert) [35]. A J-shaped curve is observed for solid cancers [36, 37] and leukemia mortality [33] in LSS. When hormesis and a downturn occur, the actual curve becomes instead an S-shaped curve [1].

### Averaging of low-dose groups

Doses < 100 mGy are the most important for our risk analyses. No significant differences were found between the control subjects and A-bomb survivors at these doses. The BEIR VII report combined all data points < 100 mGy, to which more than 80% of all survivors belong, together into one point (Fig. 4). This has been explained as an old statistical trick. It was used by Lewis to insist on the validity of LNT [21]. This dishonest representation was successful in giving the impression that the dose response is linear and that no thresholds exist. The low-dose area < 400 mGy (Fig. 4, lower left in pale green) is presented in detail (lower right in pale green) [35]. The ERR dots are dispersed widely: only 1 dot (black arrow in lower right insert) out of 12 is inside the 95% confidence interval, indicating that dose responses are not linear in this area.

### Inappropriate use of a false assumption (zero exposure-zero risk)

The line of LNT starts from zero according to the assumption that the exposure dose was zero and that ERR was zero in the control cohort (Fig. 4). This default model has been used to analyze LSS, but it is misleading because most A-bomb survivors and the control cohort people must have been exposed to residual radiation, as discussed later. The BEIR VII report based on that false assumption is therefore invalid. The dose-response line should not start from zero. Bayesian analysis does not assume this false assumption and allows more appropriate estimates. When the lower right insert of Fig. 4 is enlarged, crossing between the x-axis and the red line is roughly 25 mGy. An estimated zero dose might actually be 25 mGy. If these people were exposed to residual radiation, which was twice as great as the initial radiation, then A-bomb survivors and control subjects might have been exposed to additional 50 mGy: a total of 75 mGy.

### LNT ignores hormesis and thresholds

Granted that A-bomb survivors and control NIC people were exposed to 25–75 mGy over the estimates, the false assumption (zero exposure-zero risk) must be abandoned. Bayesian analysis, which does not need this assumption, allows negative responses, i.e., cancer mortality is suppressed to below the background level. Figure 4 shows that six responses are indeed hormetic (red dots under the x-axis in lower right insert). Therefore, low-dose radiation can suppress cancer deaths. At the same time, hormesis indicates that thresholds for positive excess risk can be established between hormetic and carcinogenic doses.

### Cherry picking of reference data

Siegel et al. [38] criticized The BEIR VII report in detail. One point is especially worthy of mention. The BEIR VII report cited that chromosomal aberrations induced by low-dose radiation in non-proliferating human cells were not repaired, thereby supporting LNT. However, that finding was a misrepresentation by failing to present that the aberrations in proliferating cells were repaired in several hours to the background level or less. Consequently, the result was opposite to what the BEIR VII argues.

## Low-dose radiation elongates A-bomb survivors’ lifespan

### Earlier studies of lifespan elongation

Stewart and Kneale [39] showed that deaths in 1950–1982 from all non-malignant diseases in LSS population were significantly lower in survivors exposed to low doses than in unexposed persons. This U-shaped dose response relationship was refuted in comments by an LSS report [40], in which the mortality of A-bomb survivors was found to fit to the linear-threshold model (the estimated threshold is 1.4 Gy (DS86)) on the basis of LNT. Mine et al. [41] and Kondo [42] analyzed total deaths among about 100,000 A-bomb survivors in Nagasaki in1970–1988 and found that 290 males exposed to 0.5–1.49 Gy (T65D) showed significantly lower mortality. Although this beneficial effect was not found in female subjects, earlier studies [39, 41, 42] hint that A-bomb survivors exposed to low to intermediate doses live longer.

### Contradiction 1: Excess relative mortality of early entrants is lower than that of late entrants

**Fig. 5:**
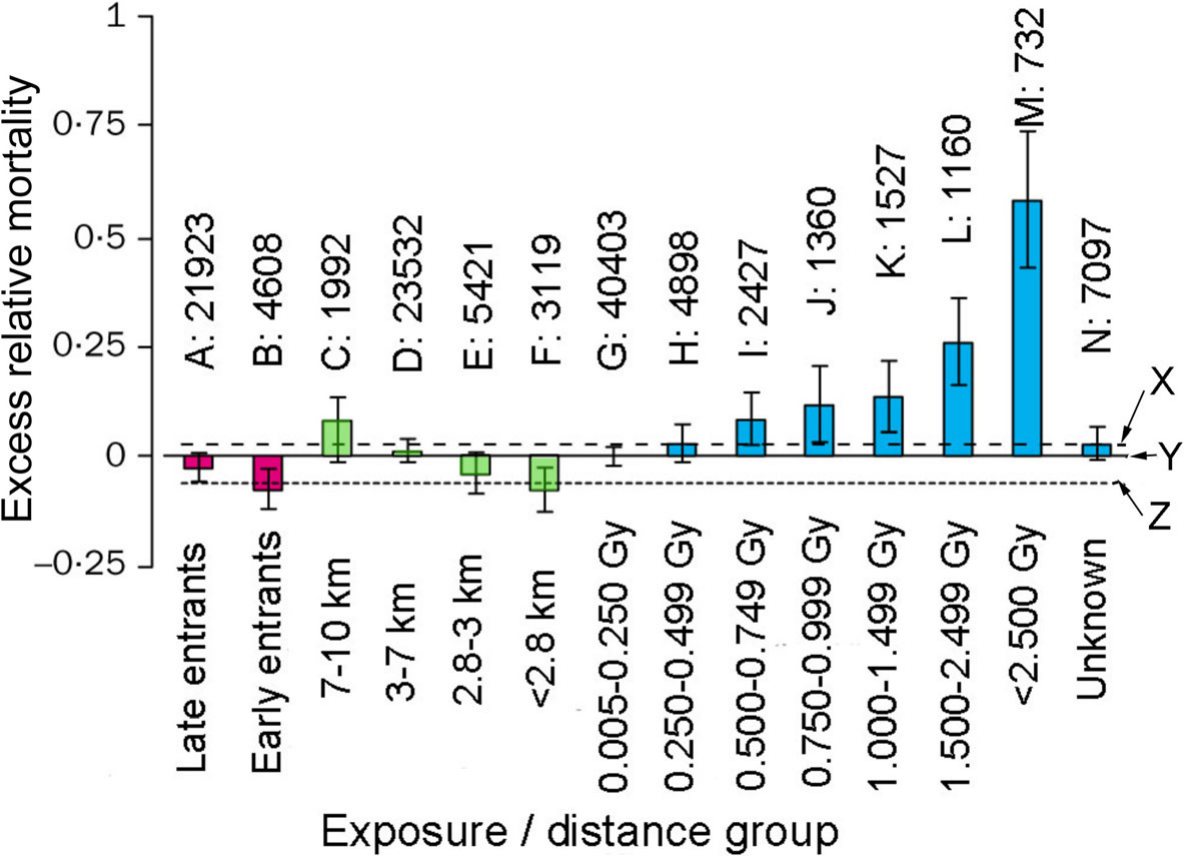
Excess relative mortality by radiation dose or distance from the hypocenter. Figure 1 and Table 1 of an earlier report [13] are combined. The scale from left to right shows increasing proximity to the epicenter: A, late entrants (not in city, entered after 1 month); B, early entrants (not in city, entered within 1 month); C–F, in city at time of A-bomb, with different distance from the epicenter; G–M, seven dose groups with different doses; and N, distance from the hypocenter = 0.11–3 km with unknown doses. Numerals above A–N denote the number of people examined. The comparison group Y (baseline mortality, or excess relative mortality 0) is all in-city individuals (*n* = 34,064) with estimated doses of zero or < 0.005 Gy. Dashed line X is the in city zero dose distal groups C and D (*n* = 25,524). Dotted line Z is the in-city zero-dose proximal groups E and F (*n* = 8540). Y is the combined data of X and Z

A-bomb survivors’ lifespans were apparently shortened as discussed earlier. Cologne and Preston’s analyses [14] were based on LNT using an assumption of zero exposure and zero risk, with no consideration of the possibility that lifespans could be elongated and that cancer deaths might be reduced. Their results are reproduced in Fig. 5.

As depicted in Fig. 3, early entrants were exposed to higher doses of residual radiation than late entrants. Excess relative mortality of early entrants, however, is lower than that of late entrants (Fig. 5A and B). The key to resolve this contradiction can be explained by radiation hormesis-related mechanisms (e.g., enhanced DNA damage repair, apoptotic removal of aberrant cells, and anticancer immunity stimulation): the B group people were exposed to higher residual radiation than the A group people. Exposure doses of the B group must be in a hormetic dose range.

### Contradiction 2: Excess relative mortality is inversely proportional to distance from the epicenter

Radiation doses are expected to be higher in proximal areas than in distal ones. If LNT is correct, then excess relative mortality must be higher in proximal areas. Data show inverse proportionality (Figs. 5C–F). Because the number of people is not small and mortality (death or life) data are accurate, the neat inverse proportionality must be close to the truth. Here again, this contradiction must be explained by radiation hormesis. People nearer the epicenter received more radiation than people farther away. Hormesis-related natural defense mechanisms also likely played a positive role in elongating the lifespan of survivors.

### Excess relative mortality shows a typical J-shaped curve, indicating hormesis and a threshold

The radiation dose group of 0.005–250 mGy (Fig. 4, G group) comprises 40,403 people. Its excess relative mortality is almost equal to that of all in-city individuals (*n* = 34,064, a total of C to F groups) whose radiation doses are estimated to be zero or < 0.005 mGy (Fig. 4, control level Y). Considering the large population size, a lack of health hazard observed in group G would not be ascribable to a simple fluctuation: it must reflect actual effects of 0–250 mGy. If they were exposed to residual radiation, which was twice as strong as the initial radiation, then they might have been exposed to additional 0–500 mGy, a total of 0–750 mGy.

The excess relative [43] mortality of H group (250–499 mGy is slightly higher than that of G group (0–250 mGy) and almost equal to D group (3–7 km from the epicenter). The mortality is below the control level X. These fluctuations are not random. At a glance from C to M in Fig. 5, one can see a beautiful J-shaped curve, an indicator of hormesis. When a J-shaped curve appears, we can establish a threshold at the crossing of the J and the x-axis. The threshold seems be between 250 and 499 mGy. Perhaps we could add 500–998 mGy of residual radiation, twice as much radiation as estimated doses.

## Cancer mortality of A-bomb survivors has been lower than the Japanese average

The US National Academy of Sciences proposed the spurious LNT in 1956 and put forward the problematic BEIR VII report in 2006 to support LNT (Fig. 4) [23]. The main reasons for the failure of the report are the use of LNT, use of the false assumption (zero exposure-zero risk), and neglect of hormesis effects. The Radiation Effects Research Foundation (RERF), a Japan–US scientific organization, has studied the health effects of A-bomb radiation. RERF has periodically reported research results and has insisted that the effects of radiation follow LNT in line with the BEIR VII report. The numbers of A-bomb survivors and solid cancer deaths are extracted from the latest three issues and are compared with Japanese averages (Table 2). The ratios of cancer deaths in both A-bomb survivors and NIC are smaller than those of Japanese averages. The numbers of people involved in Table 2 are not small. The differences are clear. Data must closely approximate reality. The finding that radiation of A-bombs reduces cancer mortality on average might be unexpected and incredible for LNT supporters. Nevertheless, such conclusions might be readily acceptable when one admits that low-dose radiation is hormetic under appropriate conditions and both A-bomb survivors and NIC who were exposed to low-dose radiation occupy a large fraction of the cohort. Consequently, low-dose radiation reduces cancer mortality on average and extends the lifespan (Fig. 5) as well.

**Table 2:** Comparison of solid cancer mortality in the lifespan study of A-bomb survivors with Japanese cancer mortality. Japanese average cancer deaths were calculated by dividing cancer deaths by total deaths each year during 1958–2009 [68]. Averages corresponding to survey periods were determined

Reporters	Year	Survey period	No. *hibakusha* or [NIC^a^]	No. cancer deaths (%)	% Japanese average cancer deaths
Preston et al. [48]	2007	1958–1998 105,427	17,448 (16.6)	21.4 (1958–1998)	21.4 (1958–1998)
			[25,4273,994	(15.7)]	
Ozasa et al. [69]	2012	1958–200	86,611	10,929 (12.6)	22.3 (1958–2003)
			[26,529	NA^b^]	
Grant et al. [70]	2017	1958–2009	80,205	17,316 (21.5)	23.3 (1958–2009)
			[25,239	5222 (20.6)]	

^a^, not in the city; ^b^, not available

## Discussion

### Earth has been exposed to ionizing radiation for billions of years

The current total heat flux from the Earth to space consists of half residual primordial heat and half radiogenic decay of uranium-238, and thorium-232, and potassium-40, the respective half lives of which are 4.46, 14.0, and 1.28 billion years [38]. Therefore, radioactivity was much higher 4 billion years ago when life started to appear on the earth. Radioactivity at our university campus in the air is less than 100 cpm, as measured with a Geiger–Muller counter, but that of nearby granite is around 500 cpm or so. Radioactive substances from the birth of the earth are still abundant on the earth now. Radon-222, a daughter of uranium-238, and radon-rich hot springs are frequently found around uranium ore.

### The human body receive roughly 20,000 radiation hits each second

In addition, carbon-14 and tritium-3 are constantly produced by the action of cosmic rays in the atmosphere. They are incorporated into our bodies. Japanese foods contain polonium-210 and potassium-40 and commit an effective dose of 0.47 mSv [44]. Consequently, the total of our annual background exposure dose is 2.1 mSv: cosmic rays (0.3 mSv), ground radiation (0.33 mSv), foods (0.99 mSv from carbon-14, polonium-210, and potassium-40), and aerial radon (0.48 mSv) [45]. When these radiation levels are converted to Bq (disintegration/second) using an Sv-Bq conversion table, rough estimation is 20,000 Bq. Potassium, an indispensable nutrient, and its associated potassium-40 (0.0117% of all naturally occurring potassium) contribute 4000 Bq. Therefore, we are exposed to by and large 20,000 radiation hits a second from not only the environment but also from materials inside our body. We ourselves are radioactive entities. In actuality, sleeping next to someone exposes one to 0.00005 mSv, which is the equivalent of eating half of a banana (0.0001 mSv). Living within 80 km of a nuclear plant and a coal plant for a year are, respectively, 0.00009 mSv and 0.0003 mSv. The dose of a chest X-ray is 0.02 mSv (ca. 1,000,000,000,000 hits [46]). A jet-liner flight from New York to London is 0.04 mSv [47]. Of course, these estimates are quite rough with significant uncertainties.

### Breathing is much more hazardous than low-dose radiation

The earth was anaerobic until 2.5 billion years ago when cyanobacteria started to add oxygen into the air. Oxygen is actually toxic, but it is useful to produce energy effectively through oxidative phosphorylation. Our ancestors started to use oxygen, but reactive oxygen species (ROS) are inescapable byproducts of the oxidative process. ROS themselves are toxic. Nine billion ROS are produced in a cell a day [48]. We developed systems to quench ROS instantaneously using radical scavengers such as glutathione and L-cysteine and using enzymes such as superoxide dismutase and catalase.

Hazards by both respiration and low-dose ionizing radiation are caused mainly by ROS, but ROS production by respiration overwhelms that by low-dose radiation by thousands to a million of times the magnitude. ROS-quenching systems developed under intensive ionizing radiation conditions for more than billion years before the appearance of oxygen in the air must be readily applied to quench ROS by respiration.

### Low-dose radiation is not only beneficial but necessary

A benefit of oxygen beyond energy production is the shielding of ultraviolet (UV) light. We sometimes expose clothes and mats to the sunlight to dry them and simultaneously kill bacteria, fungi, and ticks. We are suntanned in the sun, by which dead epithelial cells are shed from the skin when UV is strong. When oxygen was not in the air, UV was so strong that organisms were unable to live on the ground. The ozone layer cuts most UV; organisms today can move across the ground. Although UV can kill some organisms, it is indispensable to produce vitamin D. We are using the toxic UV as a need. So are ROS. When leukocytes “eat” bacteria, they enzymatically produce large quantities of ROS to kill them. ROS are sufficient to kill bacteria, but cells are also killed later. We used to see pus, a pile of dead leukocytes, in or around the wound before antibiotics became popular. In fact, J.F. Miesher extracted DNA from pus for the first time in 1869.

Figure 5 and Table 2 respectively show radiation-hormesis-related benefits: 1) elongating of lifespan and 2) reduced cancer deaths. Other analyses of LSS show hormesis in solid cancers [29] and leukemia [33]. Hormesis has been reported for many organisms such as protozoa [38], Drosophila [31, 32], and mice [49]. Lung cancer incidence of humans exposed to radon-222 is also hormetic [50]. These are some examples, constituting only the tip of the iceberg. Radiation-hormesis-related health benefits are possibly universal among all living organisms. Low-dose radiation is apparently not only beneficial but also necessary. When human cells were cultured under unshielded (1.75 mGy/y) and 10 cm lead-shielded (0.3 mGy/y) conditions, heat shock proteins (products of adaptive responses) were produced more in shielded cells than in unshielded cells, indicating that reduced radiation was not relief, but was stressful to the cells [51]. When bacteria were cultured 650 m underground, where radiation levels were 1/80 those at ground level, bacterial growth was retarded [51, 52]. If LNT is correct, then growth should be enhanced by removal of hazardous ionizing radiation. The results were the opposite, indicating the failure of LNT. Low-dose radiation is sensed by bacteria and gene expression is changed greatly at the transcriptional level [48].

### Systematically associated many-layered defense mechanisms that LNT ignores

The sanctuary zone of a 30 km radius in Chernobyl is a paradise for animals and birds. More than 315 species thrive there. Glutathione levels of rats are elevated, but no DNA lesions are found on the animals. Levels of this radical scavenger in birds of 16 species are also high [53]. The authors argue that hormesis is working there. Consequently, ROS are quenched before attacking DNA. If DNA is injured by a large amount of ROS, cells can repair most of them. If DNA injuries exceed the repair capacity, cells are killed by apoptosis and are removed. If cancerous cells are produced, then most of them are removed by vigilant survey of immune systems. These adaptive defense systems are only some examples acquired by living organisms through evolution as innate essential attributes. Humans have the ability to sense crisis and to prepare for defense. Even if ionizing radiation is neither seen nor sensed, its products, ROS, constitute signaling molecules for defense systems. Defense systems at various levels (cells, tissues, organs, etc.) by various mechanisms (ROS quenching, DNA repair, apoptosis, anticancer immunity, etc.) must be associated with hormetic dose-response relationship for radiation induced cancer. A fundamental failure of LNT is that it ignores these time-requiring biological systems. Indeed, LNT is aptly accused of “epidemiology without biology” [54].

### Magic of epidemiology to change negative to positive

A large body of experimentally obtained results collectively indicates radiation hormesis, but LNT proponents ignore these data. Risk of death from leukemia and lymphoma in more than 300,000 radiation-monitored workers (INWORKS) was studied. Results indicate that the dose-response matched well with LNT [55]. This result was praised in an internationally prestigious journal: *Nature* [56]. Soon more than 20 researchers raised objections, some of which included 1) lack of negative control, 2) LNT-based analyses, 3) no consideration of natural background and smoking, 4) 90% confidence limits (usually 95%) to achieve easy statistical significance, 5) one-tailed tests ignoring possible hormetic response, and 6) primitive miscalculations a schoolboy would not make. Soon a correction appeared in *Nature*, “The original version of this article incorrectly calculated an ‘expected’ death rate from leukaemia among the workers, and as a result, the risk posed by radiation increments was wrong. The story has been corrected to reflect this.” At least two works have leveled detailed criticisms against INWORKS studies [57, 58]. Epidemiology is apparently the last foothold for LNT, but “flexibility in data collection and analysis allows presenting anything as significant” [59]. The present author required no sophisticated epidemiology to find the opposite of what the authors assert in elongation of lifespan in Fig. 5 and a decrease of cancer mortality in Table 2.

### Tremendous human, social, and economic losses caused by obstinate application of the linear no-threshold model

The individual external doses of 421,394 Fukushima residents for the first 4 months after the 2011 earthquake and tsunami were the following: 62.0%, < 1 mSv; 94.0%, < 2 mSv; 99.4%, < 3 mSv. The arithmetic mean and maximum for individual external doses were 0.8 and 25 mSv, respectively [60]. When actual external exposure doses estimated by individual glass-badge measurements in Date City, Fukushima, were compared with official ambient doses presented by the Japanese government, the ratio was 0.15 [61]. If this figure is applied to the data above [51], then the effective doses can be calculated as follows: 62.0%, < 0.15 mSv; 94.0%, < 0.3 mSv; 99.4%, < 0.45 mSv. The respective mean and maximum doses were 0.12 and 3.75 mSv. Even the maximum external dose is below the Japanese average medical exposure dose: 4 mSv. At the time of the Fukxushima nuclear accident, the International Commission on Radiological Protection (ICRP) recommended reference levels of 20–100 mSv [62]. Less than 100 mSv, the so-called low-dose range (Fig. 4), is accepted as representing no difference between exposed and non-exposed people. These are acute doses. Hazardous effects can be reduced to 1/16.5 by prolonged radiation such as in Fukushima [63], meaning that 1.65 Sv (100 × 16.4 mSv) might be non-hazardous. If it were not for LNT, evacuation would not have been necessary in Chernobyl or Fukushima [37]. In Ramsar, Iran, people have lived continuously in environments of 260 mSv with no health problems [56]. Tremendous human, social, and economic losses caused by obstinate application of the failed LNT could have been avoided [3]. In truth, LNT is a deeply immoral. Prof. G. Walinder’s words, “The LNT hypothesis is a primitive, unscientific idea that cannot be justified by current scientific understanding. As practiced by the modern radiation protection community, the LNT hypothesis is one of the greatest scientific scandals of our time.” Madame M. Curie’s words, “Nothing in life is to be feared, it is only to be understood. Now is the time to understand more, so that we might fear less.” It is the time to reconsider the use of the LNT [64]. The author’s sincere hope is that some unmasking of LNT can help Fukushima people and others to live their lives free of irrational fear.

## Conclusion

The linear no-threshold hypothesis (LNT) was recommended without solid data by the National Academy of Sciences in 1956. The academy put forward the BEIR VII report in 2006 as supporting evidence of LNT. This report was based on the Life Span Study (LSS) of A-bomb survivors. LSS has three major defects: 1) Residual radiation to which both A-bomb survivors and control subjects were exposed was neglected. Specifically, the control subjects were not valid as representing the negative control. 2) LNT is the basis of risk analyses. The failed model cannot be used. 3) Radiation hormesis is beyond the scope of LSS, but it actually occurs. The average lifespan of A-bomb survivors is longer than the Japanese average. Solid cancer deaths of A-bomb survivors and control subjects were fewer than the Japanese average. Consequently, one can reasonably infer that radiation of A-bombs elongated their lifespan and reduced cancer deaths on average, indicating a failure of LNT. Unfortunately, LNT has served as the basis of radiation regulation. If it were not for LNT, then evacuation of Fukushima people would not have been mandated and tremendous human, social, and economic losses would have been avoided. To avoid unnecessary losses and fear, humanity must learn as soon as possible that low-dose radiation is not only harmless but beneficial.

## Abbreviations

BEAR: Biological Effects of Atomic Radiation
BEIR: Biological Effects of Ionizing Radiation
ERR: Excess relative risk
GP: Genetics Panel
ICRP: International Commission on Radiological Protection
LET: Linear energy transfer
LNT: linear no-threshold model
LSS: Life Span Study
NAS: National Academy of Sciences of the United States of America
NIC: not-in-the-city
RERF: Radiation Effects Research Foundation
RF: Rockefeller Foundation
SI: International System of Units
USA: United States of America
