# Correction to: Low-dose radiation from A-bombs elongated lifespan and reduced cancer mortality relative to un-irradiated individuals

**DOI:** 10.1186/s41021-019-0127-6

**Published:** 2019-04-19

**Authors:** Shizuyo Sutou

**Affiliations:** School of Pharmacy, Shujitsu University, 1-6-1 Nishigawara, Naka-Ku, Okayama-Shi, 703-8516 Japan

**Correction: Genes Environ.**

**https://doi.org/10.1186/s41021-018-0114-3**

In the original publication of this article [1], the author pointed out the reference list mismatched citations in the main text. All citations and references have been updated.

In Table 1, the data is in incorrect volume. The correct table is below.

**Table 1:** Comparison of solid cancer mortality in the lifespan study of A-bomb survivors with Japanese cancer mortality. Japanese average cancer deaths were calculated by dividing cancer deaths by total deaths each year during 1958–2009 [2]. Averages corresponding to survey periods were determined

Reporters	Year	Survey period	No. *hibakusha* or [NIC^a^]	No. cancer deaths (%)	% Japanese average cancer deaths
Preston et al. [3]	2007	1958–1998	105,427	17,448 (16.6)	21.4 (1958–1998)
			[25,427]	[3,994 (15.7)]	
Ozasa et al. [4]	2012	1958–200	86,611	10,929 (12.6)	22.3 (1958–2003)
			[26,529]	[NA^b^]	
Grant et al. [5]	2017	1958–2009	80,205	17,316 (21.5)	23.3 (1958–2009)
			[25,239]	[5,222 (20.6)]	

^a^not in the city; ^b^ not available

The publisher apologizes to the readers and author for the inconvenience.

The original publication has been corrected.
